# Communication during covid area: The impact of creating a new communication center

**DOI:** 10.1016/j.amsu.2022.103866

**Published:** 2022-05-25

**Authors:** Aabdi M, A. Loubaris, Motiaa Y, O. Es-Saad, S. Labib, H. Sbai

**Affiliations:** a Intensive Care Unit, Tanger Tetouan Al Hoceima University Hospital Center, Faculty of Medicine and Pharmacy, Abdelmalek Essaâdi University, Tangier, Morocco; b Simulation Center for Medical Formation, Faculty of Medicine and Pharmacy, Abdelmalek Essaâdi University, Tangier, Morocco

## Abstract

**Background:**

Since the breakout of COVID-19 pandemic, many ways have been proposed to improve the quality of communication between the medical staff patient and their families

In our department of intensive care unit in tanger Morocco and due to the lack of resources we proposed to create a communication center near the hospital to keep the families updated.

**Objectives:**

Explore the outcomes and the satisfaction of family members and intensive care unit physicians about the quality of communication through reception center during the restrictive measures imposed by COVID-19.

**Methods:**

we have conducted a satisfaction survey with sample of 100 families and 35 members of ICU physician during the period between August and December 2021.

**Results:**

We found that communications allowed families a better understanding of the clinical state of their patient, However it was inferior to the visit near bed visits, they also thought that it helps reduce the stress due to the lack of information.

**Physicians believed:**

that the communication center improved the quality of care given to the patient and helped to reduce the tension with family and increased the trust link between then and family members.

**Conclusion:**

The creation of reception center improved the relation between physicians and families and improved the quality care however it remains insufficient and other strategies should be considered including telecommunication and near bed visits specially in seriouslyç ill patients

## Highlights

• Since the breakout of COVID-19 pandemic, many ways have been proposed to improve the quality of communication between the medical stuff patient and their families.
• In our department of intensive care unit in tanger Morocco and due to the lack of resources we proposed to create a communication center neat to the hospital to keep the families updated.
• Our objective was to Explore the outcomes and the satisfaction of family members and IIntensive care unit physicians about the quality of communication through reception center during the restriction measures imposed by COVID-19.

## 1 Introduction

Coronavirus changed our daily practice of medicine, many hospitals in many different countries instaured physical restrictions to reduce the contamination risk taking over the traditional communication near bed, leading to a decrease of trust in institutions and care given to patients [[1], [2], [3], [4]].

Many solutions have been proposed to keep families update and maintain their integrity including the use of technology, telephone and vidéo communication [5,6].

In our Department we have created a new center to communicate with families and keep them updated about the clinical state of their patients.

Objectives explore impact, perspectives, attitudes and outcomes of communication in this new center in both families and intensive care units physicians.

## 2 Materials and methods

### 2.1 Settings

This study was conducted at the intensive care unit hospital at the university hospital center of Tanger Tetouan Alhoceima, it was the first experience of communication at a center in Morocco.

We enrolled 100 family members during the period between August and December 2021.

### 2.2 Data for the study analyses were collected from satisfaction survey and completed with family members and ICU physicians

The aims of this study were:

• Collection opinions of families about the communication center and its role to better understand the clinical state of the patient
• The impact of the center on the ICU physicians, the quality of care given to the patient and the psychological outcome on the staff
• General appreciation of the center from local authorities

Two satisfactions survey with 25 questions each were prepared in arabic for families and in french for physicians, including several topics: satisfaction of communication; quality of care given to the patient, quality and timing of the session, and general appreciation.

#### 2.2.1 Data analysis

The results of the survey were downloaded and analysed by EXCEL [ microsoft].

## 3 Results

### 3.1 Sessions course

The communication center was created outside the hospital, the session was conducted by 2 physicians: resident and intern, it started at 2 a.m. and lasted 5 min for each family, during which physicians explained the clinical state of the patient, the seriousness of the situation, the therapeutic projects and different possible evolution.

### 3.2 Demographic characteristics

Of 175 families, only 100 family members have agreed to participate in this survey, the mean age of family members was 45 years old, 60% of the sample were male, either partner 45% or adult child 35% All the families spoke directly to the physicians (see Table 1).

For the medical staff, 30 physicians were included 15 residents and 15 interns, the mean age was 26,5, 60% were male with average years of experience between 2 and 6 years old, (Table 2).

**Table 1:** Family member characteristics.

Family characteristics	Nombre %
Male gender	60 60%
Age: mean years	45
Relation to the patient	
Partner	50
Adult child	25
Other relation ship	25

**Table 2:** Physician characteristics.

Physician characteristics	
Mean years	26,5
Male gender	60%
Mean Years old experience	2 years old
Previous experience Covid excluded	15 50%
Previous communication study	10 33%

### 3.3 Family perspectives about communication

Families believed that communication helped them to have a better understanding on the clinical situation of the patient (70%), they expressed a better satisfaction about the quality of care given to the patient (65%), they asked to be updated about the event of any clinical worsening (80%), to be consulted before tracheal intubation (55%) and to communicate with a senior (80%).

However, family members claimed that the duration of the session was insufficient 90%, and wished they could see the patient directly (90%)

All family responses are summarized in Table 3.

**Table 3:** Perrcentage of patient satisfaction.

Questions	% of satisfaction
General appreciation of the session	65
Schedule of the session	55
The duration of the session	45
Conditions during the waiting period	65
Conditions of the reception	68
satisfaction with physician's explanations	77
Preparation before receiving bad news	80
Psychological preparation before patient discharge	70
Explanations given by doctors	65

The satisfaction survey follows LIKERT's scale [7].

The study follows PROCESS checklist [8].

### 3.4 Physicians perspectives about communication

Physicians believed that the creation of the communication center helped to reduce the tension with families 80%, improved quality of patient care 70% and reduce stress 65%

The main constraints found during the session were the risk of contaminating families 55%, the difficulty of reaching sessions at fixed schedule especially during peak periods 80%,

The physicians claims to receive inappropriate reaction from the families: verbal aggression 60%, physical aggression 20%

## 4 Discussion

Participation of families care and decision making remain, important, however, restrictive measures takenp to reduce transmission risk of Covid-1 infection, have affected the quality of communication with families [5,6,9].

The aim of a good communication is to recognise the patient and families perspectives, respect their wishes, share honest information and show empathy [3], it allows to stop the propagation of false news and information via social media, and word-to-mouth [10] the false information spread quickly and lead to social panic and may worsren the tension [11].

The miscommunication during Covid-19 has a high risk, leading to trust and credibility issues in authorities and medical staff and decrease in people trust in institutions [3,12], which make the elimination of false information and news via good communication a better way to increase decision taking and outcomes [11].

A good communication with families requires transmission of information to the person of trust designed by the patient; good listening, showing empathy and asses both patient and families requests, sharing honest information about the clinical condition in a comprehensive way, discuss possible ways of care and respect the role of families as care partners [3,5], It s also important to note that dealing with emotions is more crucial than giving a lot of technical information [13].

In our study, families thought that direct communication with medical staff has a positive impact, allowing a daily aupdate on the clinical states of the patient and evolution of the case, however they believed that the time of session was insufficient and must be lengthened, and that the schedule must be respected, and the communication team must be fixed.

Other ways of communication were used around the world; including the use of telephone and video calls [13,14]. In our context, we privileged the center over telecommunication to avoid social discrimination since a large part of the patients could not communicate via internet.

Clinicians with an experience with talking to families found this conversation familiar, however they are faced to new challenges including dealing with emotions and goodbyes especially via telecommunication [15].

A good communication by trained people helps to get better at facing grievances and suffering among families, reduce anxiety, depression, post traumatic stress, burn out and psychological distress among physicians and improve outcomes of the patients [5,15,16].

It also allows to decrease the stressful impact imposed by restriction measures [[17], [18], [19], [20]].

Our study showed that a good communication allowed young physicians to acquire a good experience in human relationships with patients and their families and improved their outcomes. However, it has a number of limitations: first the total number interviewed remain insignificant considering the number of cases in the region, and the results may not be generalized beyond our country.

## 5 Conclusion

The creation of communication center appears to be effective substitution to near bed communication, the use of a current understandable language and showing empathy are important during the communication session.

New alternatives can be proposed in the future including use of video communication and teleconference.

## Sources of funding

This research was not funded.

## Author contributions

AABDI Mohammed: Conceptualization; Data curation; Formal analysis.

LOUBARIS Alae: Conceptualization; Data curation; Formal analysis.

MOTIAA Youssef: Conceptualization; Data curation; Formal analysis.

ES-SAAD Ounci: Conceptualization; Data curation; Formal analysis.

LABIB Smael: Conceptualization; Data curation; Formal analysis.

SBAI Hicham: Supervision; Validation; Visualization; Writing – original draft; Writing – review & editing.

## Registration of research studies

Research registry 6573.

## Guarantor

AABDI Mohammed.

SBAI Hicham.

## Provenance and peer review

Not commissioned, externally peer-reviewed.

## Consent

The consent is done

## Declaration of competing interest

The authors declare no conflicts of interest.
