# Supplementary material for: Communication during covid area: The impact of creating a new communication center
Source: Ann Med Surg (Lond). 2022 May 25;78:103866. doi: 10.1016/j.amsu.2022.103866 (PMC9131702; doi:10.1016/j.amsu.2022.103866)
Supplement: Multimedia component 1 [file mmc1.docx]

PROCESS Guidelines 2020 were accepted in this paper

-       State what needs to be done next, further research with what study design (item 7b).
-       Who performed the procedures - operator experience (position on the learning curve for the technique if established, specialisation and prior relevant training (item 4h).
-       Pre-intervention considerations e.g. Patient optimisation: measures taken prior to surgery or other intervention e.g. treating hypothermia/hypovolaemia/hypotension in burns patients, ICU care for sepsis, dealing with anticoagulation/other medications and so on (item 4e)
-       Setting - describe the setting(s)and nature of the institution in which the patient was managed; academic, community or private practice setting? Location(s), and relevant dates, including periods of recruitment, exposure, follow-up, and data collection (item 4c)
-       Participants - reports numbers involved and their characteristics (comorbidities, tumour staging, smoking status, etc) (item 5a)
